# Association of Body Condition Score and Score Change during the Late Dry Period on Temporal Patterns of Beta-Hydroxybutyrate Concentration and Milk Yield and Composition in Early Lactation of Dairy Cows

**DOI:** 10.3390/ani11041054

**Published:** 2021-04-08

**Authors:** Zelmar Rodriguez, Elise Shepley, Pedro P. C. Ferro, Nilon L. Moraes, Acir M. Antunes, Gerard Cramer, Luciano S. Caixeta

**Affiliations:** Department of Veterinary Population Medicine, College of Veterinary Medicine, University of Minnesota, Saint Paul, MN 55108, USA; zrodrigu@umn.edu (Z.R.); shepl044@umn.edu (E.S.); pedropauloferro@hotmail.com (P.P.C.F.); nilonlopesmoraes@gmail.com (N.L.M.); acir_magalhaesjr@hotmail.com (A.M.A.J.); gcramer@umn.edu (G.C.)

**Keywords:** body condition score, beta-hydroxybutyrate, hyperketonemia, milk, dairy cow, transition period

## Abstract

**Simple Summary:**

In order to develop strategies to monitor and mitigate high incidences of hyperketonemia in dairy herds, the factors associated with the fluctuations of beta-hydroxybutyrate (BHB) concentration during early lactation need to be assessed. Body condition score (BCS), as well as the change in BCS during the late dry period are elements that are highly related to the mobilization of body reserves, and the proper adaptation during the transition period of dairy cattle. Our objective was to describe the pattern of blood BHB concentration and the development of hyperketonemia during the first 14 days of lactation on the basis of both a single measurement of BCS (−21 d) and the change in BCS during the late dry period. Additionally, we aimed to characterize the association between changes in BCS and milk yield and milk composition in the first monthly test. Our results suggest that changes in BCS are associated with fluctuations in BHB concentration during early lactation. In addition, we observed that cows with a loss in BCS greater than 0.5 units during the late dry period have a higher risk of having elevated BHB concentrations and incidence of hyperketonemia than cows with no change in BCS in the late dry period. Moreover, these cows also experienced lower milk production at the first monthly milk test.

**Abstract:**

Monitoring the body condition score (BCS) of dairy cows is a management strategy that can assist dairy producers in decision-making. The BCS and its variations reflect the level of body fat reserves and fat mobilization throughout the different stages of lactation. Cows that mobilize excessive amounts of fat reserves in response to the increased energy requirements of the transition period are more likely to have higher beta-hydroxybutyrate (BHB) concentration in blood, leading to a higher incidence of hyperketonemia postpartum. In this study, our main objective was to evaluate how both BCS (at 21 d prior to the expected calving date, −21 BCS) and change in BCS during the late dry period (−21 d to calving, ∆BCS) are associated with temporal patterns of blood BHB concentrations during the first two weeks of lactation. Our secondary objective was to characterize the relationship between the change in BCS in the late dry period, and milk yield and milk composition in the first milk test postpartum. In this retrospective cohort study, we assessed BCS at 21 (±3) days before the expected calving date and within three days after calving. Blood BHB concentration was measured at days 3 (±1), 7 (±1), and 14 (±1) postpartum. Hyperketonemia (HYK) was defined as blood BHB ≥ 1.2 mmol/L. To evaluate how −21 BCS and ∆BCS during the late dry period were associated with BHB in early lactation, linear mixed-effects regression models with an unstructured covariate matrix were performed. The association between ∆BCS and incidence of postpartum HYK were determined using a multivariable log-binomial model. A linear regression model was used to evaluate the association between ∆BCS and milk yield and milk composition in the first monthly test-day. Covariates used for model adjustment include parity, season, and baseline BCS. We observed that cows with BCS ≥ 4.0 at 21 d before their expected calving date had the highest BHB concentration postpartum, but no evidence that BCS ≥ 4.0 at 21 d was associated with fluctuations of BHB over time. Cows that experienced a large BCS loss (larger than 0.5 units) during the late dry period had a 61% (95% CI: 1.04, 2.50) higher risk of developing HYK in early lactation and had higher BHB concentrations during early lactation compared with cows with no ∆BCS prepartum. These associations were observed independently of the BCS at −21 d prepartum (baseline). In addition, cows that lost more than 0.5 BCS unit in the late dry period produced 3.3 kg less milk (95% CI: −7.06, 0.45) at the first milk test compared to cows that had no ∆BCS during the late dry period. No evidence of an association between −21 BCS and ∆BCS in the late dry period and milk composition was observed in our study. These results suggest that dynamic measures of BCS during the late dry period, such as ∆BCS, are better at evaluating blood BHB patterns in early lactation than BCS measured at a single time point. Cows with larger BCS loss during the late dry period and with greater parity are more likely to have higher concentrations of blood BHB postpartum, with the highest concentrations reported at 7 d post-calving.

## 1. Introduction

Body condition score (BCS) is a subjective, visual scoring system used to estimate body fat storage in cattle [1]. The change in BCS (∆BCS), rather than BCS measured at a single time point, is often used during the transition period from late pregnancy to lactation as a method of evaluating mobilization of fat reserves in dairy cattle [2,3]. The demand for energy in dairy cows during the peripartum period is elevated because of fetus growth and the concomitant onset of milk production. However, feed intake rarely matches energy requirements, leading to a period of energy deficit. Therefore, the peripartum period is characterized by increased mobilization of fat and other body reserves in an attempt to overcome the shortage of energy [4]. When fat mobilization occurs in excess, it can overwhelm the capacity of the liver to completely oxidize fatty acids. In this situation, the breakdown of fatty acids leads to an increase in the synthesis and release of ketone bodies into the bloodstream (i.e., ketogenesis). As β-hydroxybutyrate (BHB), the most stable blood ketone body, reaches concentrations ≥ 1.2 mmol/L, cows are considered to have hyperketonemia (HYK) [4,5]. The importance of HYK in the dairy industry stems from its association with reduced immune function, impaired reproductive performance, decline in milk yield, and concomitant metabolic diseases [6,7,8].

A positive association between BCS and elevated concentrations of blood BHB in early lactation has been previously described by others [9,10]. However, the relationship between both BCS 21 d from expected calving date (−21 BCS) and ∆BCS during the late dry period (i.e., three weeks before the calving date; [11]) with the fluctuations of BHB concentrations through early lactation have not yet been characterized. Furthermore, most studies evaluating ∆BCS focus on the entire dry period or on early lactation [12,13,14]. Although this is reasonable from a management perspective, considering that most diseases occur during early lactation, variations in body condition specifically during the late dry period play a fundamental role in the control of the diseases observed later in the lactation [10,15,16,17]. Therefore, a better comprehension of the influence of both −21 BCS and ∆BCS on the fluctuations of blood BHB concentrations during early lactation can be useful in the development of strategies to monitor and mitigate high incidences of hyperketonemia in dairy herds.

We aimed to assess the contribution of prepartum BCS in relation to temporal patterns of BHB concentrations during the early lactation period. Our specific objectives were to describe the temporal patterns of BHB concentration and development of HYK in early lactation on the basis of BCS, as both a single measurement and its change over the late dry period. We also aimed to characterize the relationship between change in BCS and milk yield and composition in the first monthly milk test. We hypothesized that the change in BCS during the late dry period is a better variable to evaluate the temporal patterns of blood BHB concentration during early lactation than a single BCS measurement. Additionally, a substantial loss in BCS during the dry period is expected to be associated with higher BHB concentration in early lactation and variations in milk yield and composition.

## 2. Materials and Methods

### 2.1. Study Population and Data Collection

This retrospective cohort study was performed as part of another field study evaluating an algorithm for the early prediction of HYK. Therefore, the sample size of this study can be considered as a convenience sample. The study was conducted on a commercial dairy farm located in Minnesota between February and November of 2019. The herd had 1200 lactating Holstein dairy cows, used headlocks in both dry and fresh cow pens, fed a total mixed ration, which was formulated to meet animals’ nutrient requirements, maintained reliable data records, and used a computerized data recording system (DairyPlan C21, GEA Farm Technologies, Bönen, Germany). Dry cows were housed in free-stall barns with recycled manure solid bedding and were moved to a maternity stall, bedded with straw, as soon as calving signals were identified. Lactating cows were milked three times a day and the farm participated in the Dairy Herd Improvement (DHI) program.

Our research team visited the herd three times a week for sample collection. Cows were body condition scored at two time points: −21 (±3) days (−21 BCS) and 2 (±1) days relative to calving using a 5-point scale with quarter-point increments [1]. In this scoring system, cows scored as 1 were considered emaciated, while cows scored as 5 were considered obese. The anatomical regions, considered in the measurement of BCS, included the thoracic and vertebral region of the spinal column (chine, loin, and rump), the ribs, spinous processes (loin), tuber sacral (hip or hook bones), Ischial tuberosity (pin bones), the anterior coccygeal vertebrae (tail head), and the thigh region [18]. If cows were lying in the stalls, the research team made the cows stand for better measurement. Scoring at calving was performed at least 1 day after calving to minimize the potential risk of over- or under-estimation of the BCS, measured near calving, due to the relaxation of anatomic regions (i.e., tail head ligaments). At the beginning of each visit, the research team rated the first 10 cows together to confirm scoring similarities between observers. The research team did not have access to the previous BCS when scoring BCS at calving. Parity and season were recorded at calving.

After calving, blood samples were collected immediately after morning milking at 3 (±2), 7 (±2), and 14 (±2) days in milk (DIM) to measure BHB concentration. All samples were collected from the coccygeal vessels using a 20-gauge, 2.54-cm blood collection needle and a vacuum tube with lithium-heparin (Becton Dickinson, Franklin Lakes, NJ, USA). Blood samples were immediately placed in a cooler after collection and transported on ice to the laboratory. Within 3 h of collection, samples were centrifuged at 2000× *g* for 15 min for plasma separation and kept frozen at −20 °C for later analysis. Once all samples were collected, BHB was measured in serum samples using a commercial kit colorimetric assays and an automated small-scale spectrophotometric chemistry analyzer (CataChemWell-T; Catachem Inc., Oxford, CT, USA), as described by others [19]. Intra- and inter-assay coefficient of variation of 3.9, and 13.3% respectively.

### 2.2. Statistical Analyses and Model-Building Strategies

Statistical analyses were completed in Stata version 16.0 (StataCorp, College Station, TX, USA). The study unit was the individual cow.

The −21 BCS measurement was dichotomized as moderate (BCS ≤ 3.75-reference) or overweight (BCS ≥ 4.0). The ∆BCS during the late dry period was calculated by subtracting the BCS at −21 days prepartum from the BCS measured around calving, and was categorized as gain (∆BCS range: >0 to 0.5), no change (∆BCS = 0-reference), moderate loss (∆BCS range: −0.5 to <0), or large loss (∆BCS < −0.5).

The relationship between −21 BCS and ∆BCS during the late dry period and differences in BHB concentration over the postpartum period was estimated by linear mixed-effects regression using an unstructured covariance pattern modeling [20]. Therefore, the interaction between BCS and time was evaluated. The unstructured error covariance matrix was deemed the best fit after examining a number of possible covariance matrices–including independence, exchangeable, autoregressive, and Toeplitz–based on comparison of the Akaike’s Information Criterion (AIC) score for each covariance matrix.

Due to the fact that the sample size was originally calculated for another field study, we performed a post hoc power calculation for the association between ∆BCS and BHB concentration change over time (main objective of the current study). This calculation was performed using Hotelling-Lawley Trace power calculation test, 0.05 significance level, three ordinal measurements, group size information, the observed BHB concentrations, 0.8 based correlation, and 0.1 decay between repeated measurements.

To determine the association between −21 BCS and ∆BCS prepartum and risk of hyperketonemia postpartum, we performed log-binomial regression models. Cows were considered to be hyperketonemia positive (HYK+) if the blood BHB concentrations were ≥1.2 mmol/L at any time point during the first 2 weeks postpartum, otherwise, they were considered hyperketonemia negative (HYK−).

The association between ∆BCS and milk yield (kg), energy corrected milk (kg), and fat and protein percentage at the first monthly milk test was evaluated using multivariable linear regression models and reported as mean differences. Energy corrected milk (ECM) was calculated using the following formula: ECM (kg) (3.5% fat, 3.2% protein) = (0.3246 × kg milk) + (12.86 × kg fat) + (7.04 × kg protein) [21].

Missing data of the BCS measurements (exposures) and the BHB concentrations (outcomes) were imputed performing a multiple imputations approach. Briefly, multiple imputations allow for the uncertainty about the missing data by creating several different plausible imputed data sets and combining the results from the analysis performed on each of them. We created 10 data set using the multiple imputations under the assumption of missing data at random (MAR) [22]. We tested this assumption by evaluating significant differences in BHB concentrations between available and missing data groups at each time point, concluding that the MAR assumption holds.

To account for potential confounding effects, main and effect modification (EM) models were subjected to testing for potential confounding variables. The following covariates were offered to the models: Parity (lactation =1; lactation = 2; and lactation ≥ 3) and season (spring = February–April; summer = May–July; and fall = August–October). Baseline BCS was offered as a potential confounding variable to ∆BCS model. The confounding effects were assessed, based on a 10% change in the main exposure estimate criterion [23], using a manual backward stepwise elimination procedure. Once confounding effects were assessed, biologically plausible 2-way interactions were investigated. Interactions with a *p* < 0.05 were retained in the final models.

## 3. Results

### 3.1. Descriptive Statistics

Measurements of BCS (d −21) and ∆BCS (d −21 to d 0) were obtained in 587, and 559 cows, respectively. Measurements of BHB concentration were obtained in 591, 607, and 602 cows at 3, 7, and 14 DIM, respectively. After multiple imputations, we culminated with complete data of the 637 cows originally enrolled in the study, in each of the 10 imputed datasets. As each of the 10 imputed datasets were analyzed separately–thus resulting in different, albeit similar, estimates–the reported results are presented as the average estimate of the 10 analyses. Given that only six cows gained more than 0.5 points of BCS, we decided not creating a large gain group, and remove these cows from the analyses.

The average BCS according to the categorical level of the exposure variables are presented in Table 1.

In general, moderate BCS cows gained or maintained BCS while overweight cows lost BCS during the last dry period. The descriptive statistics of each predictor by hyperketonemia group (i.e., HYK+ or HYK−) at each sampling point postpartum are presented in Table 2.

**Figure 1:**
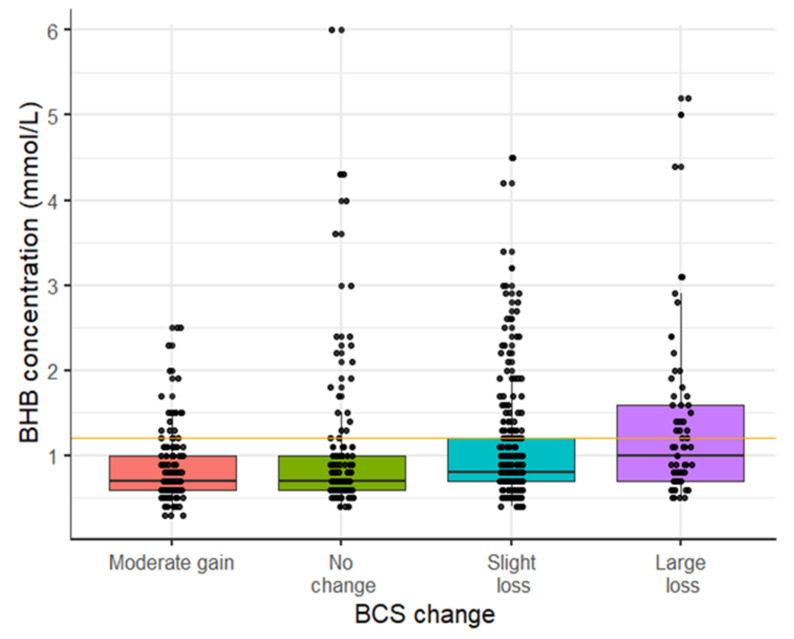
Distribution of BHB concentration during the first two weeks postpartum according to BCS change in the late dry period. Each point represents the highest BHB concentration measured during the first two weeks postpartum for each individual cow. BCS change was calculated subtracting the BCS at −21 days prepartum from the BCS measured at calving, and categorized as gain (∆BCS 0 to 0.5), no change (∆BCS = 0—reference), moderate loss (∆BCS range: −0.5 to <0), or large loss (∆BCS > −0.5). Number of cows with moderate gain = 130; no-change = 117, moderate loss = 247, and large loss = 65. The HYK incidence was for moderate gain 16.0%, no-change = 17.9%, moderate loss = 26.7%, and large loss = 17.9%.

The prevalence of HYK was 10.6%, 15.8%, and 11.9% on days 3, 7, and 14, respectively. The cumulative incidence of hyperketonemia during the two weeks postpartum was diagnosed in 24.0% (*n* = 153) of the 637 Holstein dairy cows in the study. The incidence of HYK in the first 14 days postpartum was higher among cows with some degree of BCS loss, during the late dry period, than among cows with no change or a gain in BCS in the same period (Figure 1). Primiparous cows had the lowest incidence of HYK at 4.1% followed by second parity at 25.6% and third or greater parity at 44.2%. The incidence of HYK in early lactation was 18.6% in spring, 25.0% in summer, and 30.9% in fall. The average BHB concentration across the three time points postpartum was 0.52 mmol/L for first lactation cows, 0.79 mmol/L for second lactation cows, and 0.99 mmol/L for third or greater lactation cows. Correlation between time points within-cow ranged from 0.35 (95% CI: 0.27, 0.42) to 0.46 (95% CI: 0.38, 0.53).

### 3.2. Body Condition Score 21 Days before Calving Date

**Figure 2:**
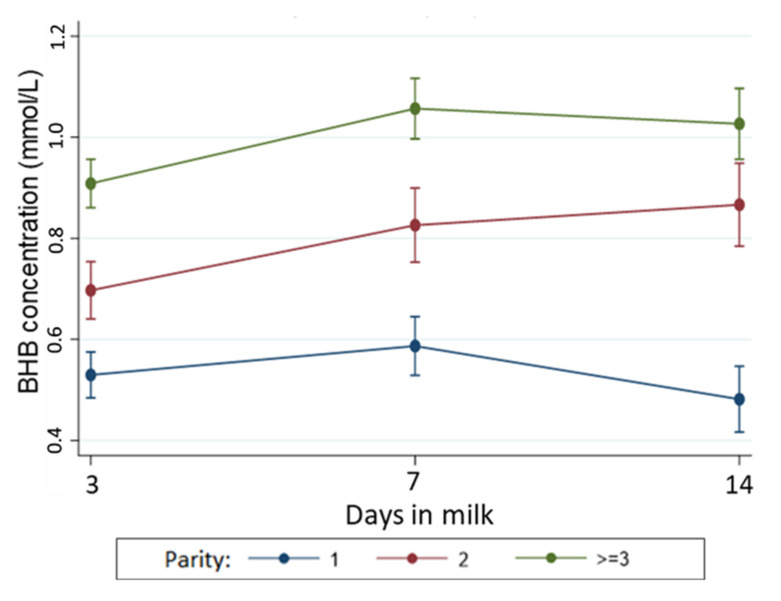
Temporal pattern of BHB concentration (with 95% confidence interval) during early lactation according to parity. The association between BHB concentrations and parity was adjusted in the model by time, BCS at 21 days prepartum, and interaction between parity and time. Number of cows with moderate gain = 130; no-change= 117, moderate loss= 247, and large loss= 65.

The incidence of HYK in the first 14 days postpartum was 14.8% (*n* = 32) for moderate (BCS ≤ 3.75) and 29.6% (*n* = 110) for overweight cows (BCS ≥ 4.0). Cows considered overweight 21 d before their expected calving date had a 63% (95% CI: 1.16, 2.28) higher risk of developing HYK than cows with moderate −21 BCS. Blood BHB concentration was, on average, 0.08 mmol/L (95% CI: 0.02, 0.14) higher in overweight cows than cows with moderate −21 BCS. However, when the interaction term of BCS and time was included (variation in BHB measurements across the sampling period), there was no evidence that −21 BCS was associated with changes in BHB concentrations over the first two weeks postpartum (*p* = 0.41). Further analyses showed evidence that the BHB concentrations across early lactation varied according to the parity of the cow (Figure 2), with BHB concentrations remaining lower in primiparous cows across all three samplings time points.

### 3.3. Change in Body Condition Score

After model adjustment, cows that experienced a large loss in BCS (∆BCS < −0.5) during the late dry period had a 61% (95% CI: 1.04, 2.50) higher risk of developing HYK in early lactation than cows with no change in BCS (∆BCS = 0) prepartum (Table 3).

**Figure 3:**
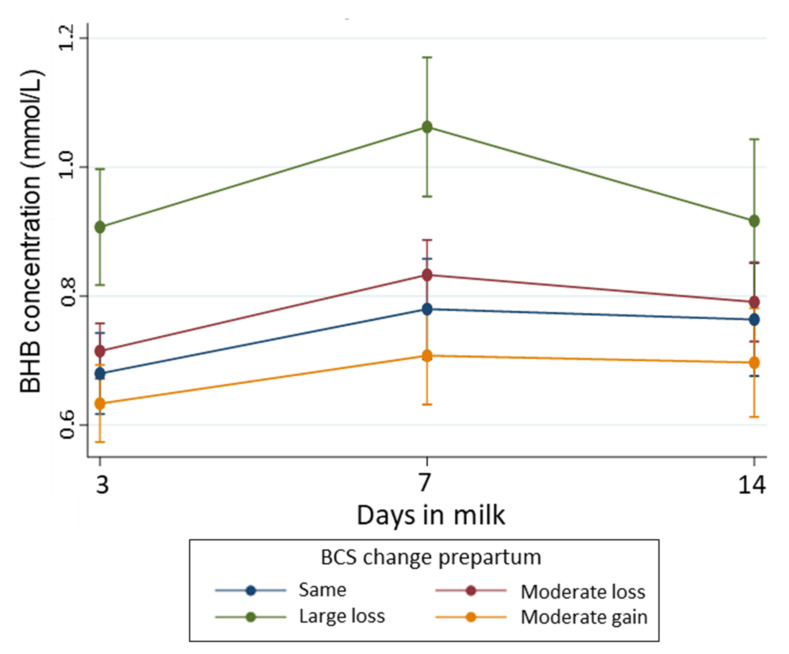
Temporal pattern of BHB concentration (with 95% confidence interval) during early lactation according to BCS change as the explanatory variable and adjusting for BCS at baseline (−21 days), parity, time, the interaction between BCS change and time, and interaction between parity and time. BCS change was calculated subtracting the BCS at −21 days prepartum from the BCS measured around calving, and categorized as gain (∆BCS 0 to 0.5), no change (∆BCS = 0—reference), moderate loss (∆BCS range: −0.5 to < 0), or large loss (∆BCS > −0.5). Number of cows with moderate gain = 130; no-change = 117, moderate loss = 247, and large loss = 65.

Cows with a moderate loss in BCS (∆BCS = −0.5 to <0) prepartum also had an increased risk of developing HYK in early lactation, but to a lower extent (RR = 1.41, 95% CI: 0.95, 2.10) than cows with large losses in BCS. Cows with a moderate gain in BCS (∆BCS = 0 to 0.5) during the late dry period had a similar risk of developing HYK as cows that had no change in BCS. Parity was a confounder in the association between ∆BCS and HYK, unlike season, which was removed from the model (Table 3). We observed that the risk of HYK increased with parity. Therefore, after controlling for ∆BCS during the late dry period, the risk of developing HYK was 88% (95% CI: 96, 82) and 75% (95%CI: 57, 17) lower for first and second parity cows, respectively, compared to third or greater parity cows. Unlike BCS measured at a single time point, ∆BCS was associated with variations of BHB concentration over early lactation. Figure 3 shows the pattern of BHB concentrations in early lactation according to ∆BCS during the late dry period. Cows that experienced a large loss in BCS during the late dry period had higher BHB concentrations during the first two weeks postpartum, with the peak BHB concentration at 7 DIM (1.07 mmol/L, 95% CI: 0.96, 1.18) and a more pronounced decline towards the end of the second week postpartum. Unlike cows with large losses in BCS, cows that experienced only a moderate loss in BCS during the prepartum period had similar BHB concentrations over time than cows with no ∆BCS and cows with a moderate gain in BCS. The post hoc power calculation for this association was 89.5%.

Table 4 shows the marginal mean and mean differences in milk production and composition during the first monthly milk test by level of BCS change during the late dry period. The results suggest that cows that experienced any degree of BCS change in any direction during the late dry period decline milk yield, compared to cows that remain the same BCS, independent of −21 BCS (baseline BCS). After adjusting milk yield by fat and protein percentage (i.e., energy corrected milk), the decline in milk yield remained only among the group of cows with a large loss in BCS. There was no evidence that protein percentage varied with variations in BCS.

## 4. Discussion

In the current study, we aimed to evaluate the dynamics of BHB concentrations in the first two weeks postpartum according to BCS at a single time point during the late dry period and the change in BCS from −21 d prepartum to calving. As a secondary objective, we aimed to evaluate whether ∆BCS during the late dry period was associated with milk yield and milk composition at the first monthly milk day test.

### 4.1. Single Measurement of BCS Prepartum

Our results suggest that there is no evidence that BCS measured at −21 d prepartum is associated with the changes in the BHB concentrations in early lactation. In addition, we observed that, on average, overweight cows (BCS ≥ 4) were more likely to have higher BHB concentrations postpartum and higher risk of HYK than cows with BCS ≤ 3.75 during the dry period. This result is in agreement with other studies using a similar dichotomization of BCS that reported a higher BHB concentration in overweight cows, with consequently increased risk of HYK [9,24]. The positive association between prepartum BCS and postpartum BHB concentrations is likely triggered by two connected factors: A decrease in the feed intake and an increase in fat mobilization [25]. Overweight cows have been shown to have a greater reduction in prepartum feed intake than cows with moderate body condition [26,27], which can lead to decreased exogenous supplies of glucose and non-carbohydrate precursors utilized in the gluconeogenesis process [28]. This situation is exacerbated by the high energy demands of the fetus and the onset of colostrum and milk production [4]. In terms of fat mobilization, previous studies have suggested that animals with excessive BCS generate higher BHB concentration as a consequence of exacerbated lipolysis [4,29]. Overweight cows have an increased stimulation of the hormone-sensitive lipase and adipose triglyceride lipase due to a higher exposure of receptors in the adipocyte [16,30,31]. Also in overweight cows, central resistance to leptin, a hormone produced by the adipocytes that induces satiety signals, can likewise lead to increases in lipolysis [32,33].

### 4.2. Change in Body Condition Score

Most of the cows in our study lost BCS during the late dry period. While 55.8% of the cows lost BCS, only 23.2% gained it. It is worth noting that changes in BCS during the late dry period may differ substantially with BCS changes during the whole dry period, by which body condition gains are usually observed [34]. The BCS changes differently through the dry period because of different physiological requirements during the early and late stages of the dry period for each individual cow [25]. For example, cows that have the same BCS at dry-off and at calving are considered to have experienced no change in BCS throughout the dry period. Although the majority of cows might have the same, or small, variations in BCS throughout the dry period, others could possibly gain BCS during the first half and lose BCS during the second half of the dry period. These cows may then be metabolically unequal. The latter might already be mobilizing adipose tissue by the time of calving in response to the metabolic challenge of the transition period. Hence, there is a loss of BCS during the late dry period, which increases the risk of developing HYK, in accordance with results described in previous studies [35,36].

Contrary to BCS assessed on a single time prepartum, ∆BCS during the late dry period was an adequate variable to describe the variability in BHB concentration during early lactation as they were associated. According to Cummings and Foster (2003) [37], deviations from the physiological, tightly regulated body fat composition trigger compensatory responses in terms satiety regulators and energy usage that persist until the level of body fat storage is restored. It has been observed that, in general, cows tend to converge on their genetically programmed BCS target in early lactation (BCS = 2.5) at 12 to 15 weeks postpartum [38]. Hence, the awareness on the relationship between BCS change and fluctuations in BHB concentration can be beneficial for on-farm hyperketonemia management purposes and other potentially energy-related issues. Examples of this would be by controlling BCS during the late dry period to avoid large losses, or by monitoring cows especially at the end of the second week postpartum.

We observed an increase in BHB concentration in the first two weeks postpartum in cows with a large loss in BCS during the late dry period. This finding is in agreement with other studies that reported a similar BHB increase in cows that lost body condition [9,10]. Rathbun et al. (2017) [9] reported an increase of BHB concentration postpartum after the loss of 1.0 units of body condition. However, in our study, the increase in BHB was observed even at losses smaller than 0.5 units of BCS. A loss in BCS is an indication that the cow mobilizes body reserves, including fatty acids from adipose tissue, in an attempt to overcome the energy deficit [39]. Cows with higher levels of circulating fatty acids are at a higher risk of developing fatty liver, as well as increased blood BHB concentration because the capacity of the liver to oxidize those fatty acids is overwhelmed [4]. Although fatty liver might influence the effect of BCS change in BHB fluctuations postpartum, other metabolic and infectious diseases around calving might also act as mediators of this association. Throughout the literature, the association between BCS change before calving and risk of dystocia or clinical mastitis are inconsistent [40,41,42], but there is clear evidence of its association with uterine infections, milk fiver or displaced abomasum [43,44].

In our study, the blood BHB concentrations of cows with larger decreases in BCS tended to increase toward the end of the first week and decrease by the end of the second week postpartum. This pattern of blood BHB concentration can be the result of the low dry matter intake and the large amount of milk produced by cows immediately after calving. During the second week, milk yield continues to gradually increase but voluntary dry matter intake tends to also increase at a faster rate than in the first week postpartum, reducing the energy deficit [45]. Similarly, Barletta et al. (2017) [46] reported an increase in BHB concentration by day seven of lactation in cows that lost body condition. This corresponds with the peak of HYK cases around the fifth day postpartum, as previously reported in the literature [47]. Conversely, cows that had a moderate gain in BCS had a similar BHB concentration and risk of HYK than cows with no change in BCS during the late dry period, independent of the initial BCS. This suggests that attempting to gain body condition to a moderate degree in the late dry period might not be necessary and that keeping the condition steady would suffice. Although we did not have a large gain BCS group in our statistical analysis (only six cows in the study gained BCS > 0.5), body condition gain over 0.5 points in the late dry period might be negative as well. Excessive BCS gains in the dry period have been associated with a higher risk of increased fat mobilization and cows that are over-conditioned at calving are more likely to suffer the negative consequences of decreased feed intake and impaired metabolic health in the peripartum, as previous discussed [48,49,50].

From our study, we can infer that cows without BCS change, or those that had moderate variations, during the late dry period, were better adapted during the periparturient period. Nevertheless, the application of measures to correct a loss of BCS during the late dry period can be challenging in practice. Ingvartsen (2006) [49] suggested that the margin in pursuing changes in BCS is limited due to the short period between dry-off and calving, and the down-regulation of appetite observed during the late dry period. According to Heuer et al. (2000) [51], this limited variation on BCS explains the inconsistent results reported by multiple studies on the association between BCS and production, reproductive performance, and health events [7,52,53]. Furthermore, Chapinal et al. (2011) [54] reported that serum metabolites (non-esterified fatty acids and BHB) provide a better insight into metabolic health status in the short-term than BCS.

### 4.3. Milk Yield and Composition

Cows that experienced variation of BCS in any direction during the late dry period produced less milk volume that cows that maintained the same BCS. The milk volume was especially lower among the cows with a larger loss in BCS. This decrease in milk yield could be the result of an insufficient energy intake during the late dry period [34]. Interestingly, cows that lost BCS did not show a significant increase in milk fat percentage at the first milk test. Generally, cows with large body fat mobilization and elevated BHB concentration are expected to have increased milk fat content than their counterparts [24,55]. Perhaps this group of cows did not lose enough BCS to generate a substantial level of fat mobilization to modify feed intake and milk fat content, or the fat mobilization was not large enough at the time that the first milk test was performed, in order to prompt a change in milk fat percentage. Ingvartsen (2006) [49] considers a mobilization of at least 40 kg of body weight (0.5 to 1.0 points of BCS) to be necessary to depress feed intake and milk yield and to increase milk fat content during the mobilization period. Conversely, cows that moderately gained BCS had a larger increase in fat percentage at the first milk test day when compared with unchanged BCS cows. Considering that milk fat can originate from two different sources–the breakdown of blood lipids (e.g., NEFA), which accounts for 40 to 60% of milk fat, and de novo synthesis within the mammary epithelial cells [45]–the observed increase of milk fat content might have been the result of a surplus of energy intake. Ohgi et al., (2005) [56] suggested that cows maintaining or gaining BCS throughout the dry period increased milk fat content as a consequence of direct utilization of NEFA by the mammary gland.

In terms of milk yield, previous studies have reported that measuring BCS, using a 5-point scale with either 0.25 and 0.50 points of increment, showed that cows with low BCS at calving (≤3.0) have increased milk yield compared to high BCS cows [38,46]. Conversely, in our study, cows with the lower BCS at calving, which are also those with the highest BCS at −21 d (Table 1) and largest body condition loss, produced less milk than cows that had no change in BCS. This was similar when adjusting by fat percentage as ECM was also lower for cows with the lower BCS at calving compared to cows with moderate BCS. A lack of adjustment by variations in BCS in the aforementioned studies [38,46], and by dry matter intake by our study, might be the source of disagreement as both factors are related to both BCS at calving and milk yield [49,56].

### 4.4. Study Limitations

In our study, only Holstein-Friesian dairy cows were included in the analyses. Due to the genetic differences between breeds in terms of fat mobilization and BHB concentration postpartum [57], extrapolation of results to other breeds needs to be done with caution.

The short follow-up period after parturition is a limitation in this study, thereby inhibiting the finding regarding which point the change in BCS would no longer affect BHB concentration. Nevertheless, it is worth noting that, even though the pattern of BHB over time was similar in all cows, those with a larger loss in BCS had higher BHB concentrations at all the time points. Another limitation of this study is the lack of information necessary to analyze and report the intra- and inter-observer agreement when assessing BCS. The inter- and intra-observer measurement of BCS have a relatively high agreement after training in BCS assessment [58]. Despite this, a certain degree of information bias on the exposure is expected. Moreover, previous studies have suggested that the use of more than one observer would have little effect on the measurement accuracy of BCS [1,3,58]. A recent study, reported that the ∆BCS over time, calculated from time points scored by multiple observers, has a moderate agreement, suggesting that a more accurate result would be obtained from the same observer doing all the measurements.

## 5. Conclusions

In the current research, we examine the association between both BCS at the onset of the dry period and change in BCS during the late dry period and the temporal patterns of BHB concentration during early lactation. We observed that the change in BCS, as a dynamic factor, is associated with variations of BHB in the first two weeks postpartum, unlike BCS measured only at −21 days relative to calving. This result suggests that, to determine how the energy deficit during the transition period affects the incidence of hyperketonemia during early lactation, changes in BCS during the dry period should be primarily considered over BCS measured at a single time point. We also observed that cows with a larger loss in BCS during the late dry period, as well as older cows are more likely to have a higher concentration of BHB and a greater incidence of hyperketonemia in early lactation. Moreover, these cows also experienced a reduction in milk at the first monthly milk test day. Our results indicate that a loss of BCS greater than 0.5 points during the late dry period, impacts on the level of BHB concentration in early lactation and milk performance of dairy cows.

## Figures and Tables

**Table 1:** Descriptive statistics for body condition score at two different points of the late dry period (−21 d and at calving), presented for the four categories of change in BCS and the overall average.

Categories of BCS Change ^1^	Observations, No. (%)	BCS Change Mean (s.d.)	BCS at −21 d ^2^, No. (%)		BCS at Calving, No. (%)	
	-	-	**Moderate ^3^**	**Overweight ^4^**	**Moderate**	**Overweight**
Moderate gain	130 (23.2)	0.28 (0.16)	89 (43.4)	41 (11.6)	50 (13.8)	130 (23.3)
No change	117 (21.0)	0 (0)	62 (30.2)	55 (15.5)	62 (17.1)	117 (20.9)
Moderate loss	247 (44.2)	−0.3 (0.13)	53 (25.9)	194 (54.8)	197 (54.3)	247 (44.2)
Large loss	65 (11.6)	−0.82 (0.17)	1(0.5)	64 (18.1)	54 (14.9)	65 (11.6)
Total	587 (100)	−0.16 (0.35)	205 (100)	354 (100)	363 (100)	196 (100)

^1^ Categories of body condition score (BCS) change between −21 to 0 days. ^2^ Body condition score at 21 days from expected calving day. ^3^ Moderate: BCS ≥ 4. ^4^ Overweight: BCS ≤ 3.75.

**Table 2:** Descriptive statistics, sample size, and chi-square test at each sampling time point postpartum.

Variable	3 DIM ^1^			7 DIM			14 DIM			Total Observations (%) ^5^
	HYK+ ^2^	HYK− ^2^	*p*-Value ^3^	HYK+	HYK−	*p*-Value	HYK+	HYK−	*p*-Value	
	[no. (%)]	[no. (%)]		[no. (%)]	[no. (%)]		[no. (%)]	[no. (%)]		
Parity	-	-	-	-	-	-	-	-	-	-
1	3 (1.3)	225 (98.7)	<0.001	6 (2.6)	223 (97.4)	<0.001	2 (0.83)	238 (99.2)	<0.001	246 (38.6)
2	12 (8.0)	137 (92.0)	-	22 (14.3)	132 (85.7)	-	23 (15.1)	129 (84.9)	-	160 (25.1)
≥3	48 (22.4)	166 (77.6)	-	68 (30.4)	156 (69.7)	-	47 (22.4)	163 (77.6)	-	231 (36.3)
Season	-	-	-	-	-	-		-	-	-
Spring	19 (10.3)	165 (89.7)	0.311	20 (11.0)	162 (89.0)	0.013	19 (10.1)	169 (89.9)	0.640	199 (31.2)
Summer	29 (9.5)	277 (90.5)	-	51 (15.8)	271 (84.2)	-	40 (12.8)	272 (87.2)	-	328 (51.5)
Fall	15 (14.8)	86 (85.2)	-	25 (24.3)	78 (75.7)	-	13 (12.8)	89 (87.3)	-	110 (17.3)
Change in BCS ^6^	-	-	-	-	-	-	-	-	-	-
Moderate gain	9 (7.32)	114 (92.7)	0.027	11 (9.3)	107 (90.7)	<0.001	9 (7.6)	109 (92.3)	0.112	130 (23.2)
No change	8 (7.1)	105 (92.9)	-	14 (12.2)	101 (87.8)	-	13 (11.5)	100 (88.5)	-	117 (21.0)
Moderate loss	22 (9.9)	201 (90.1)	-	41 (17.0)	200 (83.0)	-	32 (13.6)	203 (86.4)	-	247 (44.2)
Large loss	12 (20.3)	47 (79.7)	-	21 (32.8)	43 (67.2)	-	12 (20.0)	48 (80.0)	-	65 (11.6)
BCS −21 ^4^	-	-	-	-	-	-	-	-	-	-
Moderate	9 (4.3)	200 (95.7)	<0.001	18 (9.0)	183 (91.0)	<0.001	13 (6.4)	191 (93.6)	<0.001	216 (36.7)
Overweight	47 (14.0)	290 (86.0)	-	71 (19.8)	288 (80.2)	-	55 (15.7)	295 (84.3)	-	371 (63.3)

^1^ DIM = Days in milk. ^2^ Cows with BHB concentration ≥ 1.2 mmol/L were deemed positives (HYK+) otherwise they were considered negatives (HYK−). ^3^ *p*-Value reported for X^2^ test. ^4^ BCS at −21 days from expected calving. Cows with BCS 3–3.75 were deemed moderate; cows BCS 4–5 were considered overweight. ^5^ Observations before multiple imputations. ^6^ Change in body condition score (BCS).

**Table 3:** Association between change in BCS and HYK in early lactation, adjusted by parity.

Change in BCS ^1^	Risk	Risk Ratio	95% CI ^2^	*p*-Value
Moderate gain	0.17	0.89	0.53, 1.49	0.669
No change	0.19	Ref.	-	-
Moderate loss	0.27	1.41	0.95, 2.10	0.092
Large loss	0.32	1.61	1.04, 2.50	0.034
Parity	-	-	-	-
≥3	0.43	Ref.	-	-
2	0.25	0.59	0.43, 0.82	0.002
1	0.03	0.08	0.04, 0.17	0.000

^1^ Body condition score change between −21 to 0 days relative to the calving date. ^2^ CI = Confidence interval

**Table 4:** Variations in milk yield and composition (marginal means and mean differences) at the first monthly milk test day according to BCS-change during the close-up period (d −21 to d 0).

Change in BCS ^1^	Milk Yield (kg)		-	Energy Corrected Milk (kg)			Fat (%)		-	Protein (%)		-
	Mean	Difference in Mean (95% CI)	*p*-Value	Mean	Difference in Mean (95% CI)	*p*-Value	Mean	Difference in Mean (95% CI)	*p*-Value	Mean	Difference in Mean (95% CI)	*p*-Value
Moderate gain	37.1	−2.28 (−4.84, 0.29)	0.082	42.8	−0.58 (−3.61, 2.43)	0.940	4.61	0.29 (0.00, 0.59)	0.049	3.18	0.10 (0.00, 0.21)	0.054
No change	39.4	Ref.	-	43.3	Ref.	-	4.31	Ref.	-	3.08	Ref.	-
Moderate loss	37.4	−2.04 (−4.34, 0.25)	0.081	42.4	−0.89 (−3.60, 1.80)	0.774	4.52	0.21 (−0.05, 0.47)	0.118	3.11	0.03 (−0.05, 0.13)	0.444
Large loss	36.1	−3.30 (−7.06, 0.45)	0.084	40.4	−2.92 (−7.34, 1.49)	0.278	4.45	0.13 (−0.29, 0.56)	0.535	3.15	0.07 (−0.08, 0.23)	0.350

Models adjusted by baseline BCS and parity. ^1^ BCS measured at the calving date, and categorized as gain (∆BCS 0 to 0.5), no change (∆BCS = 0—reference), moderate loss (∆BCS range: −0.5 to < 0), or large loss (∆BCS > −0.5).

## Data Availability

The data presented in this study are available on request from the corresponding author.
